# 

**Published:** 1887-05-14

**Authors:** 


### CONTENTS.

**The Poor in Towns** - - - - - - 101

**Words of Consolation.**—XXXIII. The Work of the Holy Ghost- - - - - - 102

**Hospital Worthies.** — Dr. William Marsden, Founder of the Royal Free Hospital and Brompton Cancer Hospital - - - - - - 103

**Nursing Institutions in the Country.**—By the Rev. J. H. Timins, M.A., F.G.S. - - - - 104

**Poetry.**—The Children's Ward - - - - 106

**Incidents of Animal Life.** Collated by the Rev. F. O. Morris, B.A.—Dogs and their Doings - - 107

**Notes and News.**—Miscellaneous Items.—The Jaffa Medical Mission Hospital.—The National Pension Fund, etc., etc. - - - - - 108

**Annotations.**—Hospital Book-keeping.—Hospital Sunday.—Dynamite as a Life-Preserver - - 111

**Insanity in Child-bearing.** By Henry Rayner, M.D., Medical Superintendent, Hanwell Asylum. Part II. Other Forms of Mental Disorders - - 112

**Curiosities of Ancient Leechcraft.** By a Student of Old Books and Manuscripts. *Concluded* 113

**Questions and Answers.**—The Quilter Prize Hospital Account Forms - - - - - - - 114

**A National Pension Fund for Hospital Officials and Nurses.** The Views of Officials, Sisters, Nurses, and Others - - - - 115

**Novel or No Novel.** By Miss Louisa Twining - 115

**Till the Doctor Comes.**—XVIII. Disinfection in and after Fever Cases - - - - - - 116

**The Children's Corner.**—Puzzles, Answers, etc. - 117

**Amusements with Profit** - - - - 117

**In- and Out-Patients' Hospital Diary** - - 118

